# 3-Methyl-4-nitro­phenol

**DOI:** 10.1107/S160053681200253X

**Published:** 2012-01-25

**Authors:** Su-Lan Dong, Xiaochun Cheng

**Affiliations:** a College of Life Science and Chemical Engineering, Huaiyin Institute of Technology, Huaiyin 223003, Jiangsu, People’s Republic of China

## Abstract

In the title mol­ecule, C_7_H_7_NO_3_, the nitro group is oriented at 14.4 (3)° with respect to the plane of the benzene ring. The crystal structure is stabilized by O—H⋯O hydrogen bonds and further consolidated by C—H⋯O inter­actions.

## Related literature

For applications of the title compound in organo­phospho­rus pesticides and for the synthetic procedure, see: Yin & Shi (2005 ▶). For a related structure, see: Barve & Pant (1971 ▶).
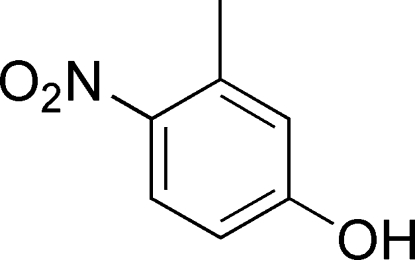


## Experimental

### 

#### Crystal data

C_7_H_7_NO_3_
*M* *_r_* = 153.14
Monoclinic, 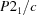

*a* = 7.2993 (14) Å
*b* = 13.023 (3) Å
*c* = 7.4445 (16) Å
β = 91.217 (4)°
*V* = 707.5 (2) Å^3^
*Z* = 4
Mo *K*α radiation
μ = 0.11 mm^−1^
*T* = 296 K
0.20 × 0.18 × 0.15 mm

#### Data collection

Enraf–Nonius CAD-4 diffractometer
Absorption correction: ψ scan (North *et al.*, 1968 ▶) *T*_min_ = 0.978, *T*_max_ = 0.983
3860 measured reflections
1293 independent reflections
1071 reflections with *I* > 2σ
*R*_int_ = 0.023
3 standard reflections every 200 reflections intensity decay: 1%

#### Refinement

*R*[*F* ^2^ > 2σ(*F* ^2^)] = 0.043
*wR*(*F* ^2^) = 0.148
*S* = 1.00
1293 reflections
102 parameters
H-atom parameters constrained
Δρ_max_ = 0.18 e Å^−3^
Δρ_min_ = −0.21 e Å^−3^

### 

Data collection: *CAD-4 Software* (Enraf–Nonius, 1985 ▶); cell refinement: *CAD-4 Software*; data reduction: *XCAD4* (Harms & Wocadlo, 1995 ▶); program(s) used to solve structure: *SHELXS97* (Sheldrick, 2008 ▶); program(s) used to refine structure: *SHELXL97* (Sheldrick, 2008 ▶); molecular graphics: *SHELXTL* (Sheldrick, 2008 ▶); software used to prepare material for publication: *SHELXTL*.

## Supplementary Material

Crystal structure: contains datablock(s) I, global. DOI: 10.1107/S160053681200253X/pv2508sup1.cif

Structure factors: contains datablock(s) I. DOI: 10.1107/S160053681200253X/pv2508Isup2.hkl

Supplementary material file. DOI: 10.1107/S160053681200253X/pv2508Isup3.cml

Additional supplementary materials:  crystallographic information; 3D view; checkCIF report

## Figures and Tables

**Table 1:** Hydrogen-bond geometry (Å, °)

*D*—H⋯*A*	*D*—H	H⋯*A*	*D*⋯*A*	*D*—H⋯*A*
O3—H3⋯O2^i^	0.82	2.00	2.811 (2)	169
C5—H5⋯O3^ii^	0.93	2.58	3.437 (2)	154

Symmetry codes: (i) 
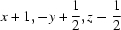
; (ii) 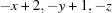.

**Fig. 1.**
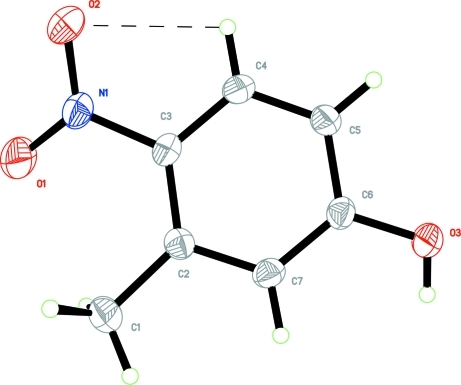
The molecular structure of the title compound with the atom numbering scheme. Displacement ellipsoids are drawn at the 50% probability level. H atoms are presented as small spheres of arbitrary radius.

**Fig. 2.**
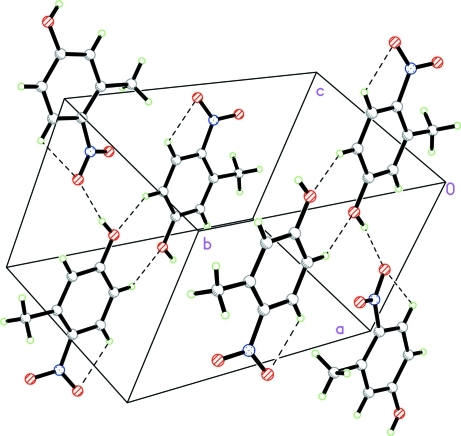
A view of the O—-H···O and C—H···O hydrogen bonds (dotted lines) in the crystal structure of the title compound.

## supplementary crystallographic information

### Comment

The tittle compound is an important intermediate which can be used in many fields such as organophosphorus pesticides (Yin & Shi, 2005).

In the title molecule (Fig. 1), the nitro group (N1/O1/O2) and the benzene ring (C2—C7) are oriented at 14.4 (3) ° with respect to each other. The molecular structure is stabilized by O3—H3···O2 intermolecular hydrogen bonds and further consolidated by C5—H5···O3 type intermolecular hydrogen bonding interactions (Tab. 2 and Fig. 2). The bond lengths and angles in the title compound are in agreement with the corresponding bond legths and angles reported for a closely related compound (Barve & Pant, 1971).

### Experimental

The title compound was prepared by a method reported in the literature (Yin & Shi, 2005). A solution of sodium nitrite (5.11 g, 74 mmol) and nitric acid (13 g, 208 mmol) in dichloromethane (50 ml) was added slowly to a solution of *m*-cresol (5 g, 46.2 mmol). After stirring for 5 h at a tempeature of 323 K, the solvent was evaporated on a rotary evaporator to obtain the title compound. The crystals were grown from an ethanol solution by slow evaporaton of the solvent at room temperature in about 7 days.

### Refinement

All H atoms were positioned geometrically and constrained to ride on their parent atoms, with O—H = 0.82 Å and C—H = 0.93 and 0.96 for aryl and alkyl H atoms, respectively, with *U*_iso_(H) = *xU*_eq_(C), where *x* = 1.2 for aryl and 1.5 for other H atoms.

### Crystal data

**Table:**

C_7_H_7_NO_3_	*F*(000) = 320
*M**_r_* = 153.14	*D*_x_ = 1.438 Mg m^−^^3^
Monoclinic, *P*2_1_/*c*	Mo *K*α radiation, λ = 0.71073 Å
Hall symbol: -P 2ybc	Cell parameters from 2201 reflections
*a* = 7.2993 (14) Å	θ = 2.8–29.8°
*b* = 13.023 (3) Å	µ = 0.11 mm^−^^1^
*c* = 7.4445 (16) Å	*T* = 296 K
β = 91.217 (4)°	Block, colorless
*V* = 707.5 (2) Å^3^	0.20 × 0.18 × 0.15 mm
*Z* = 4	

### Data collection

**Table:**

Enraf–Nonius CAD-4 diffractometer	1071 reflections with *I* > 2σ
Radiation source: fine-focus sealed tube	*R*_int_ = 0.023
graphite	θ_max_ = 25.5°, θ_min_ = 2.8°
ω/2θ scans	*h* = −8→8
Absorption correction: ψ scan (North *et al.*, 1968)	*k* = −15→15
*T*_min_ = 0.978, *T*_max_ = 0.983	*l* = −9→6
3860 measured reflections	3 standard reflections every 200 reflections
1293 independent reflections	intensity decay: 1%

### Refinement

**Table:**

Refinement on *F*^2^	Primary atom site location: structure-invariant direct methods
Least-squares matrix: full	Secondary atom site location: difference Fourier map
*R*[*F*^2^ > 2σ(*F*^2^)] = 0.043	Hydrogen site location: inferred from neighbouring sites
*wR*(*F*^2^) = 0.148	H-atom parameters constrained
*S* = 1.00	*w* = 1/[σ^2^(*F*_o_^2^) + (0.0847*P*)^2^ + 0.266*P*] where *P* = (*F*_o_^2^ + 2*F*_c_^2^)/3
1293 reflections	(Δ/σ)_max_ < 0.001
102 parameters	Δρ_max_ = 0.18 e Å^−^^3^
0 restraints	Δρ_min_ = −0.21 e Å^−^^3^

### Special details

**Table:**

Geometry. All e.s.d.'s (except the e.s.d. in the dihedral angle between two l.s. planes) are estimated using the full covariance matrix. The cell e.s.d.'s are taken into account individually in the estimation of e.s.d.'s in distances, angles and torsion angles; correlations between e.s.d.'s in cell parameters are only used when they are defined by crystal symmetry. An approximate (isotropic) treatment of cell e.s.d.'s is used for estimating e.s.d.'s involving l.s. planes.
Refinement. Refinement of *F*^2^ against ALL reflections. The weighted *R*-factor *wR* and goodness of fit *S* are based on *F*^2^, conventional *R*-factors *R* are based on *F*, with *F* set to zero for negative *F*^2^. The threshold expression of *F*^2^ > σ(*F*^2^) is used only for calculating *R*-factors(gt) *etc*. and is not relevant to the choice of reflections for refinement. *R*-factors based on *F*^2^ are statistically about twice as large as those based on *F*, and *R*- factors based on ALL data will be even larger.

### Fractional atomic coordinates and isotropic or equivalent isotropic displacement parameters (Å^2^)

**Table:**

	*x*	*y*	*z*	*U*_iso_*/*U*_eq_	
O3	1.09340 (18)	0.37232 (10)	0.0133 (2)	0.0532 (4)	
H3	1.1635	0.3301	−0.0293	0.080*	
C7	0.9309 (2)	0.21737 (13)	0.0839 (2)	0.0388 (5)	
H7	1.0295	0.1779	0.0476	0.047*	
C3	0.6322 (2)	0.23242 (13)	0.1996 (2)	0.0370 (4)	
C5	0.8008 (3)	0.38512 (14)	0.1339 (2)	0.0417 (5)	
H5	0.8101	0.4563	0.1303	0.050*	
C4	0.6461 (2)	0.33913 (14)	0.1958 (2)	0.0402 (5)	
H4	0.5497	0.3792	0.2356	0.048*	
C1	0.7737 (3)	0.05198 (14)	0.1483 (3)	0.0542 (6)	
H1A	0.7391	0.0288	0.2652	0.081*	
H1B	0.8940	0.0268	0.1226	0.081*	
H1C	0.6877	0.0266	0.0597	0.081*	
C2	0.7745 (2)	0.16801 (13)	0.1442 (2)	0.0366 (4)	
C6	0.9445 (2)	0.32372 (14)	0.0763 (2)	0.0378 (4)	
N1	0.4618 (2)	0.19093 (13)	0.2651 (2)	0.0467 (4)	
O2	0.3574 (2)	0.24929 (12)	0.3445 (2)	0.0635 (5)	
O1	0.4233 (2)	0.10112 (12)	0.2411 (3)	0.0868 (7)	

### Atomic displacement parameters (Å^2^)

**Table:**

	*U*^11^	*U*^22^	*U*^33^	*U*^12^	*U*^13^	*U*^23^
O3	0.0443 (8)	0.0454 (8)	0.0708 (10)	−0.0081 (6)	0.0235 (7)	−0.0031 (7)
C7	0.0350 (10)	0.0421 (11)	0.0396 (9)	0.0033 (7)	0.0062 (7)	−0.0029 (7)
C3	0.0335 (10)	0.0432 (10)	0.0344 (9)	−0.0041 (7)	0.0044 (7)	0.0012 (7)
C5	0.0458 (10)	0.0327 (9)	0.0470 (10)	−0.0005 (7)	0.0102 (8)	0.0002 (7)
C4	0.0369 (9)	0.0416 (10)	0.0423 (10)	0.0039 (7)	0.0085 (7)	−0.0010 (7)
C1	0.0606 (13)	0.0382 (11)	0.0643 (13)	−0.0016 (9)	0.0113 (10)	0.0002 (9)
C2	0.0409 (9)	0.0348 (9)	0.0340 (8)	−0.0015 (7)	0.0012 (7)	−0.0006 (7)
C6	0.0367 (9)	0.0396 (10)	0.0373 (9)	−0.0061 (7)	0.0069 (7)	−0.0009 (7)
N1	0.0381 (9)	0.0517 (10)	0.0507 (9)	−0.0072 (7)	0.0075 (7)	0.0021 (7)
O2	0.0437 (8)	0.0656 (10)	0.0823 (11)	0.0005 (7)	0.0253 (8)	0.0006 (8)
O1	0.0719 (12)	0.0569 (10)	0.1333 (17)	−0.0280 (9)	0.0428 (11)	−0.0163 (10)

### Geometric parameters (Å, °)

**Table:**

O3—C6	1.351 (2)	C5—C6	1.393 (3)
O3—H3	0.8200	C5—H5	0.9300
C7—C6	1.390 (3)	C4—H4	0.9300
C7—C2	1.393 (2)	C1—C2	1.511 (3)
C7—H7	0.9300	C1—H1A	0.9600
C3—C4	1.394 (3)	C1—H1B	0.9600
C3—C2	1.404 (2)	C1—H1C	0.9600
C3—N1	1.450 (2)	N1—O1	1.215 (2)
C5—C4	1.367 (2)	N1—O2	1.236 (2)
			
C6—O3—H3	109.5	C2—C1—H1B	109.5
C6—C7—C2	122.19 (15)	H1A—C1—H1B	109.5
C6—C7—H7	118.9	C2—C1—H1C	109.5
C2—C7—H7	118.9	H1A—C1—H1C	109.5
C4—C3—C2	122.34 (15)	H1B—C1—H1C	109.5
C4—C3—N1	116.23 (15)	C7—C2—C3	115.82 (15)
C2—C3—N1	121.42 (16)	C7—C2—C1	118.12 (16)
C4—C5—C6	118.99 (16)	C3—C2—C1	126.04 (16)
C4—C5—H5	120.5	O3—C6—C7	122.66 (15)
C6—C5—H5	120.5	O3—C6—C5	117.02 (16)
C5—C4—C3	120.33 (16)	C7—C6—C5	120.31 (15)
C5—C4—H4	119.8	O1—N1—O2	121.27 (16)
C3—C4—H4	119.8	O1—N1—C3	120.45 (16)
C2—C1—H1A	109.5	O2—N1—C3	118.28 (16)
			
C6—C5—C4—C3	−0.6 (3)	C2—C7—C6—O3	−178.06 (16)
C2—C3—C4—C5	1.1 (3)	C2—C7—C6—C5	1.6 (3)
N1—C3—C4—C5	−179.15 (15)	C4—C5—C6—O3	178.90 (16)
C6—C7—C2—C3	−1.0 (2)	C4—C5—C6—C7	−0.8 (3)
C6—C7—C2—C1	−179.66 (16)	C4—C3—N1—O1	165.72 (19)
C4—C3—C2—C7	−0.4 (2)	C2—C3—N1—O1	−14.6 (3)
N1—C3—C2—C7	179.95 (15)	C4—C3—N1—O2	−13.9 (2)
C4—C3—C2—C1	178.20 (17)	C2—C3—N1—O2	165.86 (17)
N1—C3—C2—C1	−1.5 (3)		

### Hydrogen-bond geometry (Å, °)

**Table:**

*D*—H···*A*	*D*—H	H···*A*	*D*···*A*	*D*—H···*A*
O3—H3···O2^i^	0.82	2.00	2.811 (2)	169
C4—H4···O2	0.93	2.35	2.671 (2)	100
C5—H5···O3^ii^	0.93	2.58	3.437 (2)	154

Symmetry codes: (i) *x*+1, −*y*+1/2, *z*−1/2; (ii) −*x*+2, −*y*+1, −*z*.
